# Initial Experience with Correlation Object–Based DRR Targeting Using Stereoscopic X-Ray Imaging in Lung SBRT

**DOI:** 10.3390/cancers18020316

**Published:** 2026-01-20

**Authors:** Marlies Boussaer, Cristina Teixeira, Kajetan Berlinger, Selma Ben Mustapha, Anne-Sophie Bom, Sven Van Laere, Mark De Ridder, Thierry Gevaert

**Affiliations:** 1 Department of Radiotherapy, Research Centre for Digital Medicine, UZ Brussel, Vrije Universiteit Brussel, 1090 Brussels, Belgium; marlies.boussaer@uzbrussel.be (M.B.);; 2 Brainlab SE, 81829 Munich, Germany

**Keywords:** SBRT, markerless, motion management, lung tumor

## Abstract

Background/Objectives: Despite significant advances in imaging technology, real-time intra-fraction monitoring of moving targets remains a challenge in markerless radiotherapy. This retrospective study investigates the use of ExacTrac Dynamic by Brainlab as an intra-fraction monitoring tool for stereotactic body radiotherapy (SBRT) in both early-stage NSCLC and oligometastatic disease. Methods: A total of 63 X-ray pairs from 21 patients were analyzed to evaluate tumor visualization with and without a surrogate approach. Statistical analysis was conducted to determine whether failures could be attributed to tumor size or localization using the Mann–Whitney U-test and Fisher’s exact test. The accuracy of the X-ray/digitally reconstructed radiograph (DRR) surrogate-based fusion was assessed by calculating and comparing the corresponding 3D vectors according to the linear mixed effects model, with a random slope effect for size of surrogate and a random intercept per patient. Results: Surrogates enhanced tumor visualization on X-ray/DRR fusions from 14.3% to 75.5%. Tumor size and lung affected (left or right) did not predict visualization success. Tumor location, however, tended to influence visibility, with lesions in the upper lobes being more readily visualized (88%) than those in the lower lobes (48.1%), although no statistical significance was reported (*p* > 0.05). Regarding geometric accuracy, 76% of the analyzed data points deviated less than 5 mm in the 3D vector measurements, the mean values were around 4 mm (±3 mm), and the medians were within 3 mm across all conditions. No statistically significant differences (*p* > 0.05) were found based on the surrogate size or the triggering time of the X-ray during the breathing cycle. Conclusions: Surrogate-based DRRs, referred to as Correlation Objects, demonstrate consistent geometric accuracy across multiple surrogate sizes and X-ray acquisitions, supporting the clinical translation of markerless lung targeting workflows for lung SBRT.

## Simple Summary

Lung tumors are rarely visible on X-rays due to their low soft-tissue contrast; however, X-rays are easy to acquire and substantially faster to acquire than conventional cone beam computed tomography. This study investigates whether a surrogate-based strategy using Correlation Objects can facilitate the use of X-rays for positioning and intra-fraction monitoring during markerless stereotactic body radiotherapy of lung tumors. Retrospective analysis of 63 X-ray pairs provides new insights into the application of surrogate-based digitally reconstructed radiographs and demonstrates promising potential for prospective use. The implementation of this surrogate-based approach could represent a significant advancement for patients eligible for stereotactic body radiotherapy of lung tumors.

## 1. Introduction

Stereotactic body radiotherapy (SBRT) is a commonly used radiation technique for treating lung lesions, including both early-stage Non-Small Cell Lung Cancer (NSCLC) and oligometastatic disease [1,2,3]. This approach is characterized by the delivery of a high, heterogeneous dose to the lesion, with a very steep dose fall-off toward the surrounding healthy tissue, administered in a small number of fractions, typically between 1 and 5 [4,5,6,7]. Due to the steep dose gradient, accurate targeting of the lesion is particularly critical: the high dose must be delivered with ultimate precision to the tumor [8]. Any misalignment, exacerbated by the interplay effect between the tumor motion and dynamic beam delivery, can compromise dose coverage and increase the risk of toxicity to surrounding healthy tissue [8,9,10]. Advances in integrated imaging techniques, also referred to as image-guided radiotherapy (IGRT), contribute to this goal; however, irradiating lung lesions remains challenging due to respiratory motion [11,12,13,14]. To account for the internal motion of the lesion, several solutions have been proposed, including tracking and amplitude, phase and/or breath-hold gating techniques, or an internal target volume (ITV) approach [15,16,17]. It is important to note that mapping the motion during simulation alone is not sufficient; continuous monitoring of the lesion’s movement throughout the radiation delivery is equally crucial to ensure optimal treatment accuracy and outcomes with SBRT [4,11,18,19].

A well-defined IGRT protocol at the time of delivery may include four-dimensional cone beam computed tomography (4D CBCT) to accurately position the patient and to visualize the motion of the tumor and its surrounding organs at risk (OARs) [15,16,20]. IGRT techniques such as stereoscopic X-rays and kilovoltage (kV) or megavoltage (MV) images can be used to monitor patient movement and make necessary corrections for any displacement caused by involuntary patient motion or drifting [11,21,22]. Intra-fractional motion management using MV imaging has the advantage that no additional radiation dose is delivered to the patient and that imaging is performed simultaneously with treatment delivery, with minimal impact on clinical workflow. A limitation of MV imaging is the lack of three-dimensional (3D) information and the fact that, for modulated treatment plans, the beam’s-eye view is not necessarily correlated with the actual tumor position or extent [23,24]. Like MV imaging, kV imaging offers significant potential for intra-fractional motion monitoring, as images can be acquired rapidly during treatment delivery. kV imaging lacks 3D information as well, and, in a markerless approach, is affected by superposition of anatomical structures such as the heart, ribs, and vertebrae, which may obscure tumor visibility. Marker-based approaches can partially overcome these limitations; however, they are associated with a substantial risk of complications due to the invasive nature of fiducial marker placement [25,26]. To address the lack of 3D information, stereoscopic X-ray imaging can be used to derive 3D tumor positioning [23,24]. Nevertheless, X-ray imaging provides insufficient soft-tissue contrast to reliably visualize lung lesions or detect potential lesion drift in a markerless approach, whereas CBCT offers superior soft-tissue differentiation [1,20]. Compared to planar X-ray, CBCT has a prolonged acquisition time and an inability to perform real-time intra-fraction motion monitoring [18]. Finally, surface guidance (SGRT) alone cannot be used for tumor localization, as a consistent correlation between external patient surface motion and internal lung tumor motion cannot always be assumed [27,28,29].

The aim of this study is to explore the possibility of visualizing tumors on stereoscopic X-ray images (ExacTrac Dynamic (ETD), Brainlab, Munchen, Germany), providing a 3D shift based on surrogates in a markerless approach. An investigation into whether the use of ETD can effectively visualize and position the lesion and monitor intra-fraction motion during SBRT for lung tumors is also conducted.

## 2. Materials and Methods

### 2.1. Patient Selection and Treatment Details

A retrospective analysis was conducted on 21 consecutive patients who received SBRT for a lung tumor. All patients were treated using an ITV approach. The following dose-fractionation schemes were applied depending on lesion location and OAR proximity: 3 × 17 Gy at the 80% isodose line; 4 × 12 Gy at the 80% isodose line; 5 × 8.5 Gy at the 85% isodose line; or 8 × 7.5 Gy at the 85% isodose line. The patients’ characteristics are presented in Table 1.

During simulation, a 4D computed tomography (4D CT) scan with 3 mm slices was performed on a GE Revolution (GE Healthcare, Chicago, WI, USA) CT scanner equipped with a respiratory gating system (RGSC; Varian Medical Systems, Palo Alto, CA, USA). Patients were asked to breathe regularly. Before the scan was performed, the patients simulated normal breathing. If necessary, the patients were coached to achieve a regular breathing pattern. The respiratory signal was captured by the RGSC system during the scan and subsequently reviewed and analyzed for irregularities. The 4D CT was used to delineate the ITV based on the 10 phases. A planning target volume (PTV) margin of 5 mm was added to account for setup uncertainties. The CT exhale phase was used for treatment planning. All patients were treated on a TrueBeam STx linac (Varian Medical Systems, Palo Alto, CA, USA) equipped with ExacTrac Dynamic. Prior to each treatment session, a 4D CBCT scan was acquired for patient positioning and motion assessment. Patient positioning was performed and a 3D CBCT was acquired to verify patient positioning. Mid-treatment control 3D CBCT was performed to analyze patient motion or lesion drifting. The patient’s respiratory signal was continuously recorded during treatment delivery on the base of the ExacTrac Dynamic SGRT 2.0.2 system. Stereoscopic X-rays were acquired during treatment at exhale phases to capture multiple phases of the respiratory cycle using routinely applied departmental imaging parameters (80 kV, 32 mAs) that have consistently demonstrated optimal image visibility in the majority of lung patients. This approach allowed for assessment of intra-fraction motion across different respiratory phases.

### 2.2. Creation of Correlation Objects (Surrogates)

**Figure 1:**
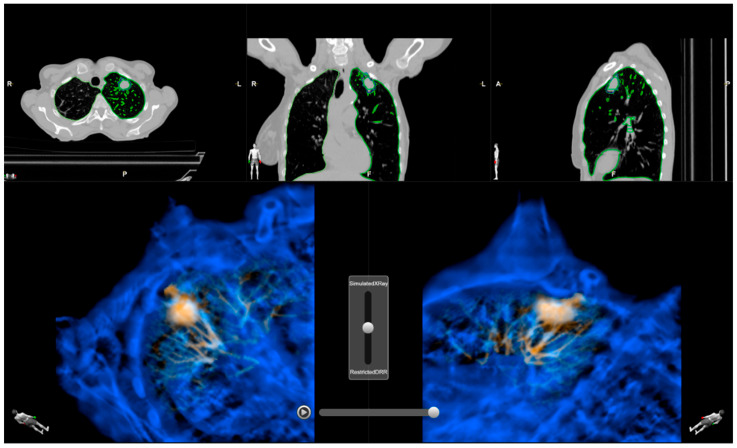

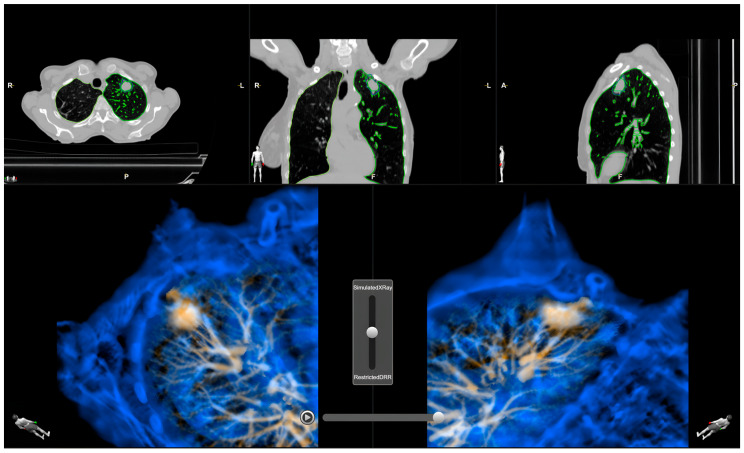
Screenshots of the pilot software “Motion Analysis” showing Correlation DRRs (amber) superimposed on simulated X-ray pairs (blue). All images rendered using the X-ray geometry of ExacTrac Dynamic. A motion margin of 0.7 mm was used at the top and 1.3 mm at the bottom.

A dedicated pilot software was developed by Brainlab SE to create anatomical surrogates for lung lesions for use in target localization within Brainlab’s ExacTrac X-ray system. These surrogates, referred to within the Brainlab SE software as Correlation Objects, are generated on a planning CT that represents a specific respiratory state, either a respiratory phase of a 4D CT or a breath-hold CT scan. The planning CT (reference CT) requires the gross tumor volume (GTV) and the affected lung lobe to be contoured. To identify respiratory motion within the affected lung lobe, at least one additional CT volume acquired at a distinctly different respiratory phase is provided to the software. An elastic registration is performed between the reference CT and the additional CT volume(s), enabling the determination of motion trajectories for the GTV and all other structures within the lung lobe. All trajectories are then compared to that of the GTV, allowing the degree of motion correlation with the lesion to be quantified for every location within the lung lobe. The software allows the definition of multiple Correlation Objects, each characterized by a specific degree of motion correlation/motion margin. The motion margin defines the maximum allowed deviation from the GTV motion trajectory used to include surrounding structures in a Correlation Object/DRR. Increasing the motion margin results in an increased object size. DRRs rendered from a selected surrogate object (Correlation DRR) can be overlaid onto simulated X-ray images in the software, enabling assessment of the suitability of a given object prior to treatment (see Figure 1 for further explanation).

For each lung lesion, three Correlation Objects were generated. Object sizes ranged from small to medium to large and was defined by the allowed motion margin around the GTV. A small surrogate was employed to achieve high localization accuracy by limiting the inclusion of peripheral structures with lower motion correlation. A medium-sized surrogate was used to increase robustness, while a large surrogate was generated to investigate deformation effects and assess their impact on localization accuracy. Each surrogate was used for retrospective localization in every acquired X-ray pair. The motion margin used to generate the surrogates ranged from 0 to 5.2 mm.

Each patient dataset included three paired biplane X-ray acquisitions at exhalation and nine corresponding DRRs generated from different surrogate configurations (three Correlation Object sizes per X-ray pair). All Correlation DRRs were reconstructed using the same underlying tumor motion trajectory, thereby representing distinct external surrogate configurations that share an identical respiratory motion tumor pattern.

### 2.3. Data Analysis

**Figure 2:**
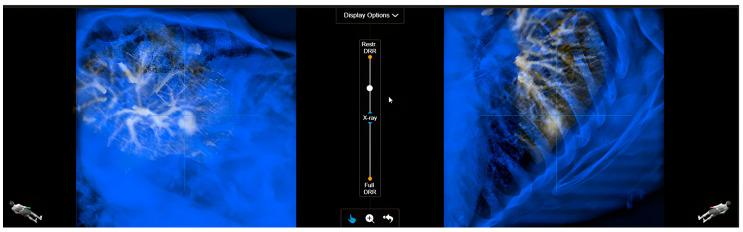
Fusion between an X-ray (blue) and a DRR containing the Correlation Object (amber). On the X-ray, multiple structures can be observed, which complicates the extraction of relevant information from the image. The Correlation Object-based DRR highlights the stable structures in the vicinity of the tumor, enabling the fusion of the two images.

Visualization of the tumor with and without a surrogate targeting strategy on an X-ray/DRR fusion was performed by a team of three experts representing the core SBRT treatment team and including a radiation oncologist, medical physicist, and dosimetrist (Figure 2).

Differences in the distribution of visualization outcomes across tumor location (upper versus lower lobe) and laterality (right versus left lung) were evaluated using Fisher’s exact test, applying a significance threshold of *p* < 0.05. Tumors were classified as “visible” if at least one visualization assessment differed from “not visible.” To examine the association between tumor size (volume in cubic centimeters) and visualization, a Mann–Whitney U test was conducted, also using a significance threshold of *p* < 0.05.

To assess the reliability of fusion and the agreement among three surrogate-based registration methods (small, medium, and large), the corresponding three-dimensional (3D) vector deviations (mm) were analyzed using a linear mixed-effects model incorporating a fixed effect for surrogate size and a random intercept per patient. A statistically significant non-zero slope was interpreted as evidence of a significant effect of surrogate size on fusion accuracy. To evaluate the repeatability and whether the timing of the X-ray pair influences the fusion outcome on a per-patient basis, intra-class correlation coefficients (ICCs) were calculated. Descriptive statistics of the 3D vector, including the mean, median, and standard deviation (SD), were also reported.

## 3. Results

### 3.1. Visibility with and Without the Use of Correlation Objects

In this paper, 63 X-ray pairs from 21 patients were fused with their corresponding DRRs, both with and without the use of surrogates. The lung lesion was visible in 9 of the 63 X-ray pairs (in 3 cases on both images and in 6 cases on one image of the pair). The creation of surrogates was not possible for 2 patients, as their 4D CT scans contained too many artifacts in the bronchi. After applying the fusion method with 3 different surrogates, the lesion could be identified in 129 out of 171 fusions (26 times on both images and 103 times on one image of a pair). In this part of the study, the emphasis was placed on the visibility or detectability of the lesion itself using Correlation Objects. This indicates that the use of surrogates increased visualization to 75.0%. While the observers were rarely able to localize the tumor itself on the X-ray images, the use of a surrogate-based DRR suggested improved inferability of the tumor position. A subdivision based on lesion location, lung affected, and tumor volume was made. A tendency suggesting that lesions in the upper lobe have a higher likelihood of visualization was observed: 88.0% in the upper lobe compared to 48.2% in the lower lobe, with a *p*-value of 0.09. No statistical relationship between the affected lung and visualization could be found (*p* = 1). The tumor size was not a predictive factor for visualization, since the Mann–Whitney U-test showed a *p*-value of 0.46.

### 3.2. Geometric Accuracy of the Surrogate Fusion

**Figure 3:**
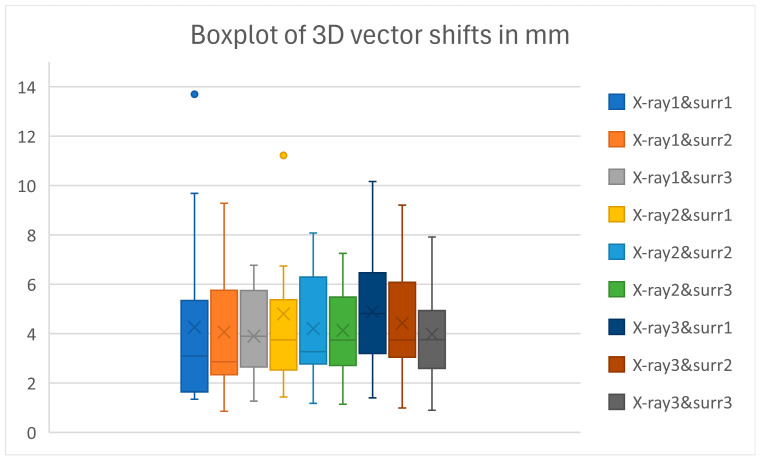
Boxplots of 3D vector shifts in mm for all subgroups.

For each of the 171 data points (19 patients with nine conditions), the 3D vector was calculated, and the results are shown in Figure 3.

From the control CBCT images, it can be inferred that 27 data points show deviating values, with larger shifts caused by patient movement. When these data points are excluded from the analysis, it can be observed that 61.1% of the remaining values exhibit a deviation smaller than 4 mm, and 75.7% a deviation smaller than 5 mm, with the 5 mm threshold chosen because it corresponds to our applied PTV margin. Results are shown in Table 2. The remaining 24.3% of data points exhibiting deviations greater than 5 mm may be attributed to inaccurate software-based matching. When the subgroup of failures is examined in detail, it can be noted that in nearly all cases, the registration is hindered by the superimposition of anatomical structures, such as the vertebrae and the heart. In one patient, it was observed that the surrogate-based DRR contained small artifacts, which could reduce the accuracy of the registration.

Overall, the mean and median 3D vectors for the 171 datapoints were 4.3 mm and 3.8 mm (SD 2.4), respectively. When 27 datapoints with a known movement or shift were excluded, the mean and median 3D vectors were 3.9 mm and 3.4 mm (SD 2.3).

### 3.3. Effect of Surrogate Size and Repeatability Across Different X-Ray Pairs on Fusion Results

The scaled residuals from the linear mixed model fit were centered around zero, with the first quartile (1Q), median, and third quartile (3Q) values of −0.3883, −0.0938, and 0.3764 mm, respectively, indicating an overall good model fit. As shown in Table 3, the fixed effects revealed an intercept of 4.862 mm (SD = 0.5973). The effect of surrogate size was estimated at −0.312 mm per mm change in surrogate size (SD = 0.202), indicating that larger surrogates tend to exhibit smaller 3D vector magnitudes. However, this effect was not statistically significant (*p* = 0.124).

Random effects analysis revealed an intercept variance of 3.941 mm^2^ (SD = 1.99 mm) between different patients and a residual variance of 2.425 mm^2^ (SD = 1.56 mm) within a patient. The intercept variance represents variability between patients, while the residual variance reflects variability within individual patients. The ICC was calculated as 0.62, indicating moderate-to-good repeatability within patients. The ICC was derived as the ratio of the random intercept variance to the total variance (sum of random intercept and residual variance).

## 4. Discussion

This study opens perspectives for the adoption of surrogate-based DRRs, enabling the visualization of small lung lesions using X-ray images. Previous data indicate that only 30% of SBRT lung tumors are visible on X-ray images [1,30]. However, when the tumor diameter increases to 3.6 cm, the probability of distinguishing the tumor on X-rays can increase up to 80% [1]. This paper investigates the potential to visualize lung lesions using surrogate-based strategies. The motion analysis tool of the Brainlab beta software can generate a surrogate, which consists of the lung lesion and its associated lung structures, such as bronchioles, moving along with the tumor within a user-defined margin, demonstrating a visibility of 14% for the lesion itself, which increases to 75% when a surrogate-based DRR is used, the latter suggesting that Correlation Objects help to infer the lesion. It is important to note that even when the lesion or surrogate is not visible to the observer, the software can still perform a reliable X-ray/DRR fusion based on the surrogates. Due to the specific configuration of the stereoscopic X-ray setup, superposition of the heart, liver, and spine remains a limiting factor; nevertheless, good and patient-specific X-ray settings (for instance, higher kV or mAs) can resolve many imperfections [26]. This study did not find a correlation between lesion size and visualization ability. However, tumors in the upper lobe are easier to visualize than those in the lower lobe. A possible explanation is that lower lobe lesions tend to move more and are subject to more artifacts, or that superposition of abdominal structures is more pronounced [14]. Further research is needed to confirm this finding.

Evaluation of the geometric accuracy of the X-ray/surrogate DRR fusion revealed no correlation with surrogate size. This finding may appear counterintuitive, as larger surrogates (up to 5.2 mm), in this study referred to as surrogate 3, might be expected to introduce greater margins of error. However, this was not observed, likely because bronchioles move in multiple directions, causing movement-related errors to be dispersed rather than systematically biased. Observers noted that DRRs based on larger surrogates may facilitate the detection of errors or provide an easier means to verify the proposed fusion results, most likely because a larger surrogate provides a more comprehensive overview. Consequently, one possible approach is to use a smaller surrogate for automatic fusion to achieve the highest possible accuracy while relying on a larger surrogate for the subsequent visual inspection of the result.

Although these results are promising, there remains room for improvement. Adjustments can be made to minimize the occurrence of fusion misalignments. For instance, a smaller slice thickness of the CT scan may produce smoother and more usable surrogates. In this study, a slice thickness of 3 mm was used, and for some surrogate structures this thickness could be clearly distinguished. While a smaller slice thickness would likely result in smoother structures and improved fusion results, the actual clinical impact of these observed failures remains to be evaluated and should be addressed in a prospective study. Additionally, the use of a deep inspiration breath hold (DIBH) CT instead of a 4D CT bin, as applied in this study, can further reduce motion artifacts, thereby enabling the creation of higher-quality surrogate-based DRRs [17,31]. Patient-specific kV and mAs X-ray settings could also improve the fusion process [32]. To test the robustness of this technique, a specific surrogate size was compared across all acquired X-rays. No significant difference was observed. This is encouraging, as it suggests that the fusion result is indeed stable.

These results are consistent with previous reports evaluating ExacTrac Dynamic and similar markerless tracking systems [1,21]. Buschman et al. [33] demonstrated the feasibility of surface and thermal correlation for intra-thoracic targets, while Bertholet et al. [18] validated combined infrared and X-ray tracking for dynamic targets. Klein et al. [34] compared DRR–X-ray matching in CyberKnife and reported comparable geometric precision. Unlike prior phantom-based studies, this work analyzed clinical patient data, capturing intersubject variability in respiratory patterns and anatomical changes. The observed reproducibility across surrogates reinforces the concept that DRRs derived from correlated surrogates can serve as reliable motion surrogates for markerless lung targeting. Limitations include the modest sample size per group and the retrospective design. These aspects will be addressed in an ongoing prospective clinical trial designed to validate the clinical feasibility and reproducibility of markerless X-ray/DRR registration using ExacTrac Dynamic in lung SBRT.

These innovative surrogate-based strategies will bring substantial benefits to the treatment of SBRT in lung cancer patients. While intra-fraction monitoring of lesions without markers has proven to be challenging [1,9,15], this technique could offer a potential solution. Ideally, combining specific motion management techniques, such as DIBH with this method of fusion, could have a significant impact on patient outcomes. Positioning would no longer need to rely on the time-consuming 4D CBCT or a 3D gated CBCT. An X-ray would represent a significant time saving compared to a CBCT. The longer the patient positioning takes, the greater the likelihood of intra-fraction motion [18,20]. Additionally, X-rays could be taken during irradiation to monitor intra-fraction motion. This would track both the patient’s motion and the movement of the lung lesion during treatment. Brainlab’s software is capable of both mapping surface scanning for patient movement and using X-rays to monitor internal tumor motion during radiation. Combining this with a DIBH technique would not only provide substantial time savings but also significantly reduce the impact on at-risk organs and decrease the risk of possible toxicity [31]. Further research on this approach is needed to validate this assumption. Nevertheless, these preliminary data open the door to effective intra-fraction motion monitoring using X-ray images for SBRT lung cancer treatment.

## 5. Conclusions

X-ray/DRR registration using surrogate-based reconstructions, referred to as Correlation Objects, showed consistent accuracy across multiple surrogates and X-ray acquisitions. These findings support the clinical translation of markerless lung targeting workflows and motivate prospective evaluation in a multi-patient trial setting.

## Figures and Tables

**Table 1:** Patients’ characteristics.

SBRT Lung Patients				
Patient ID	Schema	Left/Right	Lobe	Volume (cc)
UZB_01	5 × 8.5 Gy	Left	Lower	2.19
UZB_02	5 × 8.5 Gy	Left	Upper	1.75
UZB_03	4 × 12 Gy	Left	Upper	7.84
UZB_04	8 × 7.5 Gy	Right	Upper	4.81
UZB_05	8 × 7.5 Gy	Right	Upper	26.43
UZB_06	8 × 7.5 Gy	Right	Upper	18.31
UZB_07	4 × 12 Gy	Left	Upper	0.38
UZB_08	5 × 8.5 Gy	Left	Upper	2.98
UZB_09	8 × 7.5 Gy	Left	Lower	3.15
UZB_10	5 × 8.5 Gy	Right	Upper	0.72
UZB_11	4 × 12 Gy	Right	Upper	0.82
UZB_12	5 × 8.5 Gy	Right	Upper	1.72
UZB_13	5 × 8.5 Gy	Left	Lower	2.53
UZB_14	4 × 12 Gy	Right	Upper	1.22
UZB_15	5 × 8.5 Gy	Left	Lower	1.09
UZB_16	4 × 12 Gy	Right	Lower	8.21
UZB_17	5 × 8.5 Gy	Left	Upper	0.80
UZB_18	5 × 8.5 Gy	Right	Lower	1.23
UZB_19	5 × 8.5 Gy	Left	Lower	0.13
UZB_20	8 × 7.5 Gy	Right	Upper	1.37
UZB_21	8 × 7.5 Gy	Left	Upper	11.27

**Table 2:** Three-dimensional vector of all nine conditions. All underlined values were known to be shifted due to patient motion (surr = surrogate).

	Size (mm)			3D Vector								
	surr1	surr2	surr3	X-ray1 & surr1	X-ray1 & surr2	X-ray1 & surr3	X-ray2 & surr1	X-ray2 & surr2	X-ray2 & surr3	X-ray3 & surr1	X-ray3 & surr2	X-ray3 & surr3
UZB_01	2.5	3.5	4.5	9.679	9.280	6.214	11.220	6.710	6.789	10.160	6.075	5.941
UZB_02	1.1	1.5	2.5	5.340	5.500	4.704	5.201	4.278	6.308	6.532	6.956	2.247
UZB_03	0.5	0.7	0.9	1.889	2.502	4.195	2.163	3.263	3.738	2.581	2.629	3.965
UZB_04	1.4	1.9	2.4	5.001	2.335	2.796	5.372	3.002	4.349	4.365	6.518	7.912
UZB_05	0.4	0.6	0.8	1.435	2.587	2.900	3.750	2.968	2.939	6.661	1.967	1.806
UZB_06	1.5	1.8	2.4	NA	NA	NA	NA	NA	NA	NA	NA	NA
UZB_07	0.7	1.1	1.4	2.223	2.302	2.650	2.205	1.473	2.943	1.459	3.489	3.410
UZB_08	2	2.7	3.3	5.278	7.382	6.769	4.361	7.165	4.506	4.805	5.874	5.847
UZB_09	0.3	0.5	0.8	13.699	7.046	5.783	17.965	6.282	5.474	9.274	4.537	4.784
UZB_10	1	1.5	2	1.533	3.976	4.443	2.526	8.073	4.837	6.466	9.204	3.758
UZB_11	0.7	1	2	6.914	6.832	5.931	6.739	7.729	7.247	6.214	6.164	6.117
UZB_12	1.5	1.8	2.2	1.640	1.375	1.269	3.184	2.371	2.617	3.415	3.226	3.480
UZB_13	4	4.5	5.2	4.818	3.987	3.895	2.594	2.773	2.625	3.197	3.184	3.180
UZB_14	1.4	1.8	2.3	5.847	5.753	5.746	5.070	4.650	4.812	5.523	5.195	4.928
UZB_15	2	3	4	NA	NA	NA	NA	NA	NA	NA	NA	NA
UZB_16	1.5	2.3	3.3	4.958	5.438	5.258	5.772	6.285	6.522	5.195	5.158	4.877
UZB_17	0.5	0.8	1.3	3.095	2.634	2.782	2.579	3.865	2.709	3.015	2.417	2.272
UZB_18	1.4	2.2	3.3	1.715	2.860	2.102	4.343	3.271	3.641	5.192	3.744	4.330
UZB_19	2	3	4.5	2.941	1.752	1.913	1.517	1.487	1.497	3.228	3.644	2.594
UZB_20	0.5	0.9	1.4	1.634	2.818	3.445	3.132	3.048	3.724	4.094	3.043	3.342
UZB_21	0.7	1.3	1.9	1.342	0.854	1.319	1.435	1.175	1.140	1.396	0.990	0.894

**Table 3:** Linear mixed model fit for random and fixed effects.

Variable	Beta Estimate	SE (Beta)	T-Value		*p*-Value
Intercept	4.862	0.597	8.139		-
Size surrogate	−0.312	0.202	−1.545		0.124
RANDOM EFFECTS					
		Variance (mm^2^)		SD (mm)	
Random intercept variance (between patients)		3.941		1.985	
Residual variance (within patient)		2.425		1.557	

## Data Availability

Data are contained within the article.

## Abbreviations

The following abbreviations are used in this manuscript:

SBRT	Stereotactic body radiotherapy
NSCLC	Non-Small Cell Lung Cancer
IGRT	Image-guided radiotherapy
ITV	Internal target volume
OAR	Organ at risk
4D CBCT	Four-dimensional cone beam computed tomography
MV	Megavolt
kV	Kilovolt
3D	Three-dimensional
ETD	ExacTrac Dynamic
PTV	Planning target volume
4D CT	Four-dimensional computed tomography
RGSC	Respiratory gating system for scanners
GTV	Gross target volume
DRR	Digitally reconstructed radiograph
ICC	Intra-class correlation coefficients
SD	Standard deviation
